# Supplementary material for: Psychosocial Wellbeing of Schoolchildren during the COVID-19 Pandemic in Berlin, Germany, June 2020 to March 2021
Source: Int J Environ Res Public Health. 2022 Aug 16;19(16):10103. doi: 10.3390/ijerph191610103 (PMC9407732; doi:10.3390/ijerph191610103)
Supplement: Supplementary file 1 [file ijerph-19-10103-s001.zip › BECOSS_psych_forpubpl_20220809.html]

Analysis report - Psychosocial wellbeing of school children during the COVID-19 pandemic in Berlin, Germany, June 2020 to March 2021


Code 

- Show All Code
- Hide All Code

# Analysis report - Psychosocial wellbeing of school children during the COVID-19 pandemic in Berlin, Germany, June 2020 to March 2021

Stefanie Theuring 1,\*; Welmoed van Loon 1; Franziska Hommes 1; Norma
Bethke 2; Marcus A. Mall 3; Tobias Kurth 4; Joa-chim Seybold 2; Frank P.
Mockenhaupt 1

1 Institute of Tropical Medicine and International Health, Charité -
Universitätsmedizin Berlin, corporate member of Freie Universität Berlin
and Humboldt-Universität zu Berlin, Germany 2 Medical Directorate,
Charité - Universitätsmedizin Berlin, corporate member of Freie
Universität Berlin and Humboldt-Universität zu Berlin, Germany 3
Department of Pediatric Respiratory Medicine, Immunology and Critical
Care Medicine, Charité – Univers tätsmedizin Berlin, corporate member of
Freie Universität Berlin and Humboldt-Universität zu Berlin, Germany 4
Institute of Public Health, Charité - Universitätsmedizin Berlin,
corporate member of Freie Universität Berlin and Humboldt-Universität zu
Berlin, Germany \* Correspondence: Stefanie.theuring@charite.de; Tel.: +49 30 450
565767

```
dt_all <- fread("data_becoss_psychosocial_forpubl.csv")
```

# Fear of CoV infection - Composit

I.e., the participant reported more than moderate fear of infection
at least once of the time points.

```
dt_temp1 <- dt_all %>%
  filter(event_Tx == "T4") %>%
  # because stepAIC cant handle NAs by itself, select relevant variables and omit NAs:
  dplyr::select(fear_infect_max_2cats_01,
                age, sex, 
                ses_income_hh_2cats_b, ses_edu_hh_2cats, 
                ses_parents_german_hh, hh_siblings_yn, 
                gad7_score_hherw_max_2cats,
                gad7_score_max) %>% 
  na.omit()

# Backwards selection model:
gm1.full <- glm(fear_infect_max_2cats_01 ~ ., data = dt_temp1, family = "binomial")
stepAIC(gm1.full, trace = F) %>% summ(exp = T)
```

|  |  |
| --- | --- |
| Observations | 135 |
| Dependent variable | fear\_infect\_max\_2cats\_01 |
| Type | Generalized linear model |
| Family | binomial |
| Link | logit |

|  |  |
| --- | --- |
| χ²(1) | 4.18 |
| Pseudo-R² (Cragg-Uhler) | 0.04 |
| Pseudo-R² (McFadden) | 0.02 |
| AIC | 179.79 |
| BIC | 185.60 |

|  | exp(Est.) | 2.5% | 97.5% | z val. | p |
| --- | --- | --- | --- | --- | --- |
| (Intercept) | 0.24 | 0.04 | 1.55 | -1.50 | 0.13 |
| age | 1.16 | 1.00 | 1.35 | 2.01 | 0.04 |
|  |
| --- |
| Standard errors: MLE |

```
# Bootstrap the backwards selection, based on AIC:
set.seed(1234)
fit.boot.aic.1 <- boot.stepAIC(gm1.full, dt_temp1, B = 1000, direction = "backward", verbose = F)
fit.boot.aic.1
```

```
## 
## Summary of Bootstrapping the 'stepAIC()' procedure for
## 
## Call:
## glm(formula = fear_infect_max_2cats_01 ~ ., family = "binomial", 
##     data = dt_temp1)
## 
## Bootstrap samples: 1000 
## Direction: backward 
## Penalty: 2 * df
## 
## Covariates selected
##                             (%)
## age                        62.1
## ses_parents_german_hh      44.0
## hh_siblings_yn             42.6
## ses_income_hh_2cats_b      33.2
## gad7_score_max             30.3
## gad7_score_hherw_max_2cats 24.1
## sex                        21.3
## ses_edu_hh_2cats           19.5
## Null                        1.4
## 
## Coefficients Sign
##                                        + (%) - (%)
## age                                    99.52  0.48
## ses_parents_german_hhb_no              97.50  2.50
## gad7_score_max                         95.38  4.62
## sexmale                                77.46 22.54
## ses_edu_hh_2catsb_lower                54.87 45.13
## gad7_score_hherw_max_2catsno_anx_sympt 10.79 89.21
## ses_income_hh_2cats_bb_0-5000           2.41 97.59
## hh_siblings_ynyes                       0.47 99.53
## 
## Stat Significance
##                                          (%)
## age                                    67.95
## hh_siblings_ynyes                      60.56
## gad7_score_max                         53.47
## ses_income_hh_2cats_bb_0-5000          50.90
## ses_parents_german_hhb_no              49.09
## gad7_score_hherw_max_2catsno_anx_sympt 46.47
## ses_edu_hh_2catsb_lower                37.44
## sexmale                                36.62
## 
## 
## The stepAIC() for the original data-set gave
## 
## Call:  glm(formula = fear_infect_max_2cats_01 ~ age, family = "binomial", 
##     data = dt_temp1)
## 
## Coefficients:
## (Intercept)          age  
##     -1.4454       0.1511  
## 
## Degrees of Freedom: 134 Total (i.e. Null);  133 Residual
## Null Deviance:       180 
## Residual Deviance: 175.8     AIC: 179.8
## 
## Stepwise Model Path 
## Analysis of Deviance Table
## 
## Initial Model:
## fear_infect_max_2cats_01 ~ age + sex + ses_income_hh_2cats_b + 
##     ses_edu_hh_2cats + ses_parents_german_hh + hh_siblings_yn + 
##     gad7_score_hherw_max_2cats + gad7_score_max
## 
## Final Model:
## fear_infect_max_2cats_01 ~ age
## 
## 
##                           Step Df    Deviance Resid. Df Resid. Dev      AIC
## 1                                                   126   170.7197 188.7197
## 2           - ses_edu_hh_2cats  1 0.009395142       127   170.7291 186.7291
## 3                        - sex  1 0.172582685       128   170.9017 184.9017
## 4 - gad7_score_hherw_max_2cats  1 0.363330458       129   171.2650 183.2650
## 5             - gad7_score_max  1 0.756199055       130   172.0212 182.0212
## 6      - ses_income_hh_2cats_b  1 0.743502736       131   172.7647 180.7647
## 7      - ses_parents_german_hh  1 1.089072528       132   173.8538 179.8538
## 8             - hh_siblings_yn  1 1.932855590       133   175.7867 179.7867
```

# Anxiety - Composit

I.e., the participant had a GAD7-score ≥5 at least once of the time
points.

```
dt_temp2 <- dt_all %>%
  # just take one time point, the respective data is the same for each T:
  filter(event_Tx == "T4") %>%
  # because stepAIC cant handle NAs by itself, select relevant variables and omit NAs:
  dplyr::select(gad7_score_max_2cats_01,
                age, sex, 
                ses_income_hh_2cats_b, ses_edu_hh_2cats, 
                ses_parents_german_hh, hh_siblings_yn, 
                gad7_score_hherw_max_2cats,
                fear_infect_max_2cats) %>% 
  na.omit()

# Full model:
gm2.full <- glm(gad7_score_max_2cats_01 ~ ., data = dt_temp2, family = "binomial")

# Backwards selection:
stepAIC(gm2.full, trace = F) %>% summ(exp = T)
```

|  |  |
| --- | --- |
| Observations | 135 |
| Dependent variable | gad7\_score\_max\_2cats\_01 |
| Type | Generalized linear model |
| Family | binomial |
| Link | logit |

|  |  |
| --- | --- |
| χ²(5) | 22.22 |
| Pseudo-R² (Cragg-Uhler) | 0.20 |
| Pseudo-R² (McFadden) | 0.12 |
| AIC | 175.67 |
| BIC | 193.10 |

|  | exp(Est.) | 2.5% | 97.5% | z val. | p |
| --- | --- | --- | --- | --- | --- |
| (Intercept) | 0.26 | 0.03 | 2.25 | -1.22 | 0.22 |
| age | 1.13 | 0.97 | 1.32 | 1.61 | 0.11 |
| sexmale | 0.57 | 0.27 | 1.19 | -1.50 | 0.13 |
| ses\_income\_hh\_2cats\_bb\_0-5000 | 2.22 | 1.04 | 4.73 | 2.06 | 0.04 |
| hh\_siblings\_ynyes | 0.56 | 0.26 | 1.18 | -1.53 | 0.13 |
| gad7\_score\_hherw\_max\_2catsno\_anx\_sympt | 0.34 | 0.15 | 0.79 | -2.53 | 0.01 |
|  |
| --- |
| Standard errors: MLE |

```
# Bootstrap the backwards selection, based on AIC:
set.seed(1234)
fit.boot.aic.2 <- boot.stepAIC(gm2.full, dt_temp2, B = 1000, direction = "backward", verbose = F)
fit.boot.aic.2
```

```
## 
## Summary of Bootstrapping the 'stepAIC()' procedure for
## 
## Call:
## glm(formula = gad7_score_max_2cats_01 ~ ., family = "binomial", 
##     data = dt_temp2)
## 
## Bootstrap samples: 1000 
## Direction: backward 
## Penalty: 2 * df
## 
## Covariates selected
##                             (%)
## gad7_score_hherw_max_2cats 86.4
## ses_income_hh_2cats_b      75.5
## sex                        57.1
## age                        54.0
## hh_siblings_yn             54.0
## fear_infect_max_2cats      41.1
## ses_edu_hh_2cats           19.9
## ses_parents_german_hh      18.2
## 
## Coefficients Sign
##                                             + (%)  - (%)
## ses_income_hh_2cats_bb_0-5000              100.00   0.00
## age                                         99.26   0.74
## fear_infect_max_2catsmaessig_to_sehr_stark  98.54   1.46
## ses_parents_german_hhb_no                   76.37  23.63
## ses_edu_hh_2catsb_lower                     35.18  64.82
## hh_siblings_ynyes                            0.56  99.44
## sexmale                                      0.18  99.82
## gad7_score_hherw_max_2catsno_anx_sympt       0.00 100.00
## 
## Stat Significance
##                                              (%)
## gad7_score_hherw_max_2catsno_anx_sympt     83.56
## ses_income_hh_2cats_bb_0-5000              78.81
## sexmale                                    66.73
## hh_siblings_ynyes                          63.70
## age                                        62.04
## fear_infect_max_2catsmaessig_to_sehr_stark 57.42
## ses_parents_german_hhb_no                  35.71
## ses_edu_hh_2catsb_lower                    34.17
## 
## 
## The stepAIC() for the original data-set gave
## 
## Call:  glm(formula = gad7_score_max_2cats_01 ~ age + sex + ses_income_hh_2cats_b + 
##     hh_siblings_yn + gad7_score_hherw_max_2cats, family = "binomial", 
##     data = dt_temp2)
## 
## Coefficients:
##                            (Intercept)                                     age  
##                                -1.3486                                  0.1265  
##                                sexmale           ses_income_hh_2cats_bb_0-5000  
##                                -0.5684                                  0.7968  
##                      hh_siblings_ynyes  gad7_score_hherw_max_2catsno_anx_sympt  
##                                -0.5842                                 -1.0711  
## 
## Degrees of Freedom: 134 Total (i.e. Null);  129 Residual
## Null Deviance:       185.9 
## Residual Deviance: 163.7     AIC: 175.7
## 
## Stepwise Model Path 
## Analysis of Deviance Table
## 
## Initial Model:
## gad7_score_max_2cats_01 ~ age + sex + ses_income_hh_2cats_b + 
##     ses_edu_hh_2cats + ses_parents_german_hh + hh_siblings_yn + 
##     gad7_score_hherw_max_2cats + fear_infect_max_2cats
## 
## Final Model:
## gad7_score_max_2cats_01 ~ age + sex + ses_income_hh_2cats_b + 
##     hh_siblings_yn + gad7_score_hherw_max_2cats
## 
## 
##                      Step Df   Deviance Resid. Df Resid. Dev      AIC
## 1                                             126   162.3021 180.3021
## 2 - ses_parents_german_hh  1 0.04623815       127   162.3484 178.3484
## 3      - ses_edu_hh_2cats  1 0.08243473       128   162.4308 176.4308
## 4 - fear_infect_max_2cats  1 1.24249122       129   163.6733 175.6733
```

# Health-related quality of life - at T4

Standardized questionnaire for HRQoL.

```
dt_temp3 <- dt_all %>%
  # just take one time point, the respective data is the same for each T:
  filter(event_Tx == "T4") %>%
  # because stepAIC cant handle NAs by itself, select relevant variables and omit NAs:
  dplyr::select(KS_T2cats_prim_sec_comb_01, 
                age, sex, 
                ses_income_hh_2cats_b, ses_edu_hh_2cats, ses_parents_german_hh, hh_siblings_yn, 
                gad7_score_hherw_2cats, 
                fear_infect_2cats,
                freetime_sports_comb_2cats, freetime_friends_comb_2cats) %>% 
  na.omit()

# Full model:
gm3.full <- glm(KS_T2cats_prim_sec_comb_01 ~ ., data = dt_temp3, family = "binomial")

# Backwards selection:
stepAIC(gm3.full, trace = F) %>% summ(exp = T)
```

|  |  |
| --- | --- |
| Observations | 103 |
| Dependent variable | KS\_T2cats\_prim\_sec\_comb\_01 |
| Type | Generalized linear model |
| Family | binomial |
| Link | logit |

|  |  |
| --- | --- |
| χ²(2) | 6.15 |
| Pseudo-R² (Cragg-Uhler) | 0.08 |
| Pseudo-R² (McFadden) | 0.04 |
| AIC | 139.12 |
| BIC | 147.02 |

|  | exp(Est.) | 2.5% | 97.5% | z val. | p |
| --- | --- | --- | --- | --- | --- |
| (Intercept) | 0.57 | 0.26 | 1.29 | -1.34 | 0.18 |
| ses\_income\_hh\_2cats\_bb\_0-5000 | 2.13 | 0.90 | 5.05 | 1.71 | 0.09 |
| freetime\_sports\_comb\_2cats>=3x woche | 0.52 | 0.23 | 1.16 | -1.60 | 0.11 |
|  |
| --- |
| Standard errors: MLE |

```
# Bootstrap the backwards selection, based on AIC:
set.seed(1234)
fit.boot.aic.3 <- boot.stepAIC(gm3.full, dt_temp3, B = 1000, direction = "backward", verbose = F)
fit.boot.aic.3
```

```
## 
## Summary of Bootstrapping the 'stepAIC()' procedure for
## 
## Call:
## glm(formula = KS_T2cats_prim_sec_comb_01 ~ ., family = "binomial", 
##     data = dt_temp3)
## 
## Bootstrap samples: 1000 
## Direction: backward 
## Penalty: 2 * df
## 
## Covariates selected
##                              (%)
## ses_income_hh_2cats_b       63.0
## fear_infect_2cats           53.9
## ses_edu_hh_2cats            47.7
## age                         46.5
## freetime_sports_comb_2cats  46.4
## freetime_friends_comb_2cats 36.6
## gad7_score_hherw_2cats      36.4
## ses_parents_german_hh       23.9
## hh_siblings_yn              23.5
## sex                         22.3
## Null                         0.3
## 
## Coefficients Sign
##                                         + (%) - (%)
## fear_infect_2catsmaessig_to_sehr_stark 100.00  0.00
## ses_income_hh_2cats_bb_0-5000           99.52  0.48
## ses_edu_hh_2catsb_lower                 97.90  2.10
## ses_parents_german_hhb_no               80.33 19.67
## hh_siblings_ynyes                       80.00 20.00
## sexmale                                 65.92 34.08
## freetime_friends_comb_2cats>=1x woche    7.10 92.90
## gad7_score_hherw_2catsno_anx_sympt       3.85 96.15
## age                                      1.94 98.06
## freetime_sports_comb_2cats>=3x woche     1.72 98.28
## 
## Stat Significance
##                                          (%)
## ses_income_hh_2cats_bb_0-5000          73.02
## fear_infect_2catsmaessig_to_sehr_stark 69.20
## ses_edu_hh_2catsb_lower                62.05
## freetime_friends_comb_2cats>=1x woche  60.38
## freetime_sports_comb_2cats>=3x woche   59.91
## age                                    55.91
## gad7_score_hherw_2catsno_anx_sympt     53.02
## ses_parents_german_hhb_no              47.28
## sexmale                                40.81
## hh_siblings_ynyes                      40.00
## 
## 
## The stepAIC() for the original data-set gave
## 
## Call:  glm(formula = KS_T2cats_prim_sec_comb_01 ~ ses_income_hh_2cats_b + 
##     freetime_sports_comb_2cats, family = "binomial", data = dt_temp3)
## 
## Coefficients:
##                          (Intercept)         ses_income_hh_2cats_bb_0-5000  
##                              -0.5542                                0.7545  
## freetime_sports_comb_2cats>=3x woche  
##                              -0.6631  
## 
## Degrees of Freedom: 102 Total (i.e. Null);  100 Residual
## Null Deviance:       139.3 
## Residual Deviance: 133.1     AIC: 139.1
## 
## Stepwise Model Path 
## Analysis of Deviance Table
## 
## Initial Model:
## KS_T2cats_prim_sec_comb_01 ~ age + sex + ses_income_hh_2cats_b + 
##     ses_edu_hh_2cats + ses_parents_german_hh + hh_siblings_yn + 
##     gad7_score_hherw_2cats + fear_infect_2cats + freetime_sports_comb_2cats + 
##     freetime_friends_comb_2cats
## 
## Final Model:
## KS_T2cats_prim_sec_comb_01 ~ ses_income_hh_2cats_b + freetime_sports_comb_2cats
## 
## 
##                            Step Df   Deviance Resid. Df Resid. Dev      AIC
## 1                                                    92   126.3149 148.3149
## 2                         - sex  1 0.03303545        93   126.3479 146.3479
## 3              - hh_siblings_yn  1 0.20091422        94   126.5488 144.5488
## 4       - ses_parents_german_hh  1 0.25084853        95   126.7997 142.7997
## 5 - freetime_friends_comb_2cats  1 0.82362396        96   127.6233 141.6233
## 6      - gad7_score_hherw_2cats  1 0.71977558        97   128.3431 140.3431
## 7                         - age  1 1.38523199        98   129.7283 139.7283
## 8            - ses_edu_hh_2cats  1 1.48682415        99   131.2151 139.2151
## 9           - fear_infect_2cats  1 1.90159950       100   133.1167 139.1167
```
